# EG-VEGF maternal levels predict spontaneous preterm birth in the second and third trimesters in pregnant women with risk factors for placenta-mediated complications

**DOI:** 10.1038/s41598-023-46883-6

**Published:** 2023-11-14

**Authors:** Tiphaine Raia-Barjat, Céline Chauleur, Constance Collet, Florence Rancon, Pascale Hoffmann, Morgane Desseux, Nicolas Lemaitre, Mohamed Benharouga, Antoine Giraud, Nadia Alfaidy

**Affiliations:** 1 Department of Gynecology and Obstetrics, Hôpital Nord, University Hospital, Centre Hospitalier Universitaire de Saint-Étienne, Avenue Albert Raimond, 42270 Saint-Étienne, Saint Priest en Jarez France; 2 INSERM U1059 SAINBIOSE, Université Jean Monnet, Saint-Étienne, France; 3 Institut National de La Santé et de la Recherche Médicale (INSERM), U1292, MAB2 Team, Laboratoire de BioSanté, Bât C3, Pièce 304B.17 rue des Martyrs, 38054 Grenoble, France; 4 Commissariat à l’Energie Atomique (CEA), DSV-IRIG, Grenoble, France; 5 Université Grenoble Alpes (UGA), Grenoble, France; 6 INSERM, Centre d’Investigation Clinique 1408, Saint-Étienne, France; 7 Neonatal Intensive Care Unit, Centre Hospitalier Universitaire de Saint-Étienne, Saint-Étienne, France

**Keywords:** Health care, Disease prevention

## Abstract

Prediction of spontaneous preterm birth in asymptomatic women remains a great challenge for the public health system. The aim of the study was to determine the informational value of EG-VEGF circulating levels for prediction of spontaneous preterm birth in the second and third trimesters in pregnant women at high risk for placenta-mediated complications. A prospective multicenter cohort study including 200 pregnant patients with five-serum sampling per patient. Women with spontaneous preterm birth have higher concentrations of serum EG-VEGF than uncomplicated patients at 24 weeks, 28 weeks and 32 weeks (p = 0.03, 0.02 and < 0.001). The areas under the curve reached 0.9 with 100% sensitivity at 32 weeks for the prediction of spontaneous preterm birth. Serum EG-VEGF concentrations could be considered as a reliable biomarker of spontaneous preterm birth in high-risk for placenta-mediated complications pregnant women.

## Introduction

Preterm birth (PTB) is defined as all births occurring before 37 weeks of gestation (WG) by the Women Health Organization^1^. Preterm birth concerns 9.9% of livebirths worldwide in 2020^2^. Spontaneous preterm birth (sPTB), due to spontaneous onset of labor represents 40–45% of PTB and premature rupture of membranes (PROM) represents 25–30% of preterm births^3^. To date, sPTB is the first cause of neonatal mortality and morbidity^4,5^. The risk factors for sPTB are, a prior preterm birth, black race, periodontal disease, and low maternal body-mass index^3^. Prediction of sPTB in asymptomatic women remains a great challenge for the public health system. A short cervical length and a raised cervical-vaginal fetal fibronectin concentration are predictors of sPTB but their performance remains very low^6^. Effective and safety screening tools are still not available in clinical practice, such as the use of amniocentesis-based predictive risk models that are still under investigations^7,8^. Early identification of women who will exhibit sPTB will allow intensification of the patient monitoring, vaginal progesterone medications, pessary and/or cervical cerclage placement, and decision taking for antenatal corticosteroid therapy.

Prokineticins (PROK) are secreted peptides with a capacity to control both angiogenic and inflammatory processes in humans and in other species^9–11^. The PROK family accounts two members, PROK1 and PROK2^9^. The canonical member of this family, PROK1, is also known as Endocrine Gland-derived Vascular Endothelial Growth Factor (EG-VEGF). PROKs act through specific G protein-coupled receptors, PROK receptor 1 (PROKR1) and PROK receptor 2 (PROKR2) to control multiple biological functions such as, angiogenesis, circadian rhythm, neurogenesis of olfactory bulb, neuronal survival, reproduction, and inflammation^11^. EG-VEGF and PROKR1 are highly expressed in first trimester and in term placenta and fetal membranes^11–14^. Several studies reported increased levels of EG-VEGF and its direct involvement with its receptors in the etiology of recurrent pregnancy loss, gestational trophoblastic diseases and pregnancy pathologies such as fetal growth restriction and preeclampsia^15,16^. These data strongly suggested that the increase in EG-VEGF levels may either contribute to the development of Placenta-Mediated Pregnancy Complications (PMC) or rather participates into the overall compensatory mechanism that occur to allow the pregnancy to progress.

In relation to preterm delivery and parturition, reports from the group of Jabbour^17–20^ and from our group^12,14^ strongly suggested that deregulations in the levels of expression of members of the prokineticin family, including their receptors may be associated with the etiology of preterm and term births. In 2014, we demonstrated that circulating EG-VEGF and its expression in the fetal membranes increased towards term and significantly decreased at the time of labor^12^. In addition, the expression of its receptors exhibited the same profile towards term and an abrupt decrease at the time of labor^12^. Altogether, these results strongly suggested that EG-VEGF is a new cytokine that may act locally to ensure uterine quiescence during the third trimester of pregnancy. We believe that EG-VEGF’s contribution to the initiation of human labor is exhibited by the abrupt decrease in its levels and those of its receptors.

These findings highlight the role of EG-VEGF and its receptors as key actors that ensure quiescence of the intrauterine tissues in late pregnancy and further our understanding on their potential association with the physiopathology of preterm pregnancies. Nevertheless, no prospective study involving women at high-risk pregnancies has been conducted to determine whether EG-VEGF levels could be considered as indicators of a subsequent occurrence of preterm birth.

The objective of this study was to determine the concentrations of EG-VEGF in the plasma of pregnant women at high risk for placenta-mediated complications in the second and third trimesters for the prediction of spontaneous preterm birth and the perspective of considering the usefulness of EG-VEGF as a new biomarker of sPTB.

## Material and methods

### Study design and population

The study is based on the analyses of data collected from the AngioPred clinical trial, as previously described^21^. This AngioPred is a prospective multicenter cohort study conducted between June 2008 and October 2010 in the Obstetrics and Gynecology department of Saint Etienne and Nimes University Hospitals, and the Laboratory of Hematology in Nimes University Hospital. The initial aim of this cohort study was to evaluate several biomarkers for predicting the occurrence of PMC. The patients included in this study had consulted within 20 weeks, and were all at high risk for occurrence or recurrence of PMC. The risks included, diabetes, chronic hypertension, obesity, maternal age younger than 18 years or older than 38 years, chronic kidney disease, systemic lupus erythematosus, antiphospholipid syndrome, family history of cardiovascular disease or venous thromboembolism in first degree relatives, biological thrombophilia without any personal history of venous thromboembolism or of PMC, a history of one or more episodes of PMCs or personal history of venous thromboembolism. The exclusion criteria were the following; twin pregnancies, patients with a history of fetal death, IUGR which etiology was of chromosomal, genetic or infectious origin, and the presence of any PMC or venous thromboembolism at inclusion.

The Ethics Committee and Institutional Review Board of the University Hospital of Saint Etienne approved the protocol in March 2008. The study was registered on clinicaltrials.gov (identifier NCT00695942). The clinical investigation was performed according to the Helsinki Declaration of 1975, as revised in 1996. All patients were included before 20 weeks of gestation and gave their written consent. Informed consent was obtained from all women.

### Blood collection

Blood samples were collected at the collection center of the University Hospital of Saint-Étienne and Nîmes at 20, 24, 28, 32, and 36 weeks of gestation, totaling 5 samples per patient. The samples were immediately sent to laboratories for analysis. The samples were centrifuged, aliquoted, and stored at − 80 °C.

### Biological analysis

Each analysis was performed in blind manner to other analyses. All samples from the same patient were grouped in the same series of assays. Analyses were carried out after defrosting for 10 min in a water bath at 37 °C and centrifugation at 2500*g*. Serum EG-VEGF levels were measured at 20, 24, 28, 32, and 36 weeks by an enzyme-linked immunosorbent assay (ELISA) kit (PeproTech, Neuilly-Sur-Seine, France) with a standard range between 16 and 1000 pg/mL. Two separate standard curves were constructed to allow accurate readings of samples at the upper and lower ranges of the assays.

### Outcomes

The primary outcome was the occurrence of spontaneous preterm birth, defined as the number of spontaneous commencement of labor with intact or pre-labor rupture of membranes and birth at or after 20 weeks and 0 days of gestation, and before 37 weeks and 0 days of gestation.

### Statistical analysis

Statistical analyses were performed using XlSTAT®. Qualitative data were presented as absolute and relative frequencies (expressed in %). The qualitative variables were compared by the Chi-square test or by Fisher's exact test if the numbers were insufficient. Quantitative variables were presented as mean and standard deviation. Normal distribution of data was tested with the Shapiro–Wilk test. Results were reported as boxplots. The threshold value of EG-VEGF plasma levels for the prediction of spontaneous preterm birth was determined at each gestational age through the receiver operator characteristics (ROC) curve, calculating the area under the curve with 95% confidence intervals (95% CI)^22^. All hypotheses tests were performed at the 0.05 significance level; as such, p < 0.05 was considered significant.

## Results

### Clinical characteristics

Between June 2008 and October 2010, 200 consecutive pregnant women were included in the study. Demographics and inclusion criteria are summarized in Table 1. During the study, 45 women had a PMC and were excluded from the analysis. Seven women presented a spontaneous preterm birth between 31 and 36 weeks, 6 days. All demographic characteristics and inclusion criteria were similar between uncomplicated women and women with sPTB.

**Table 1:** Patient characteristics at inclusion.

	No complication N = 148	Spontaneous preterm birth N = 7	P value
Maternal characteristics			
Age, years (SD)	31.5 (4,9)	28.3 (6.5)	0.95
Parity, mean (SD)	1.2 (1.0)	1.4 (1.0)	0.51
BMI (Kg/m^2^)	25.0 (5.9)	29.0 (12.0)	0.75
Smoking	17 (11.5)	2 (28.6)	0.20
Maternal history			
Diabetes	5 (3.4)	0 (0)	0.62
Kidney disease	3 (2.0)	0 (0)	0.70
Chronic hypertension	8 (5.4)	0 (0)	0.52
Lupus	11 (7.4)	2 (28.6)	0.06
Antiphospholipid syndrome	5 (3.4)	0 (0)	0.62
Personal history of VTE	28 (18.9)	3 (42.9)	0.13
Personal history of PMC	89 (60.1)	4 (57.1)	**0.01**
Family history of VTE or cardiovascular disease	32 (21.6)	1 (14.3)	0.62

Categorical variables reported as frequency (percentage) and continuous variables reported as mean (standard deviation).

*BMI* body mass index, *MAP* mean arterial pressure, *Mean UARI* mean uterine artery resistance index, *VTE* venous thromboembolism, *PMC* placenta-mediated complication.

Significant values are in bold.

### Serum EG-VEGF levels and the occurrence of spontaneous preterm birth

**Figure 1:**
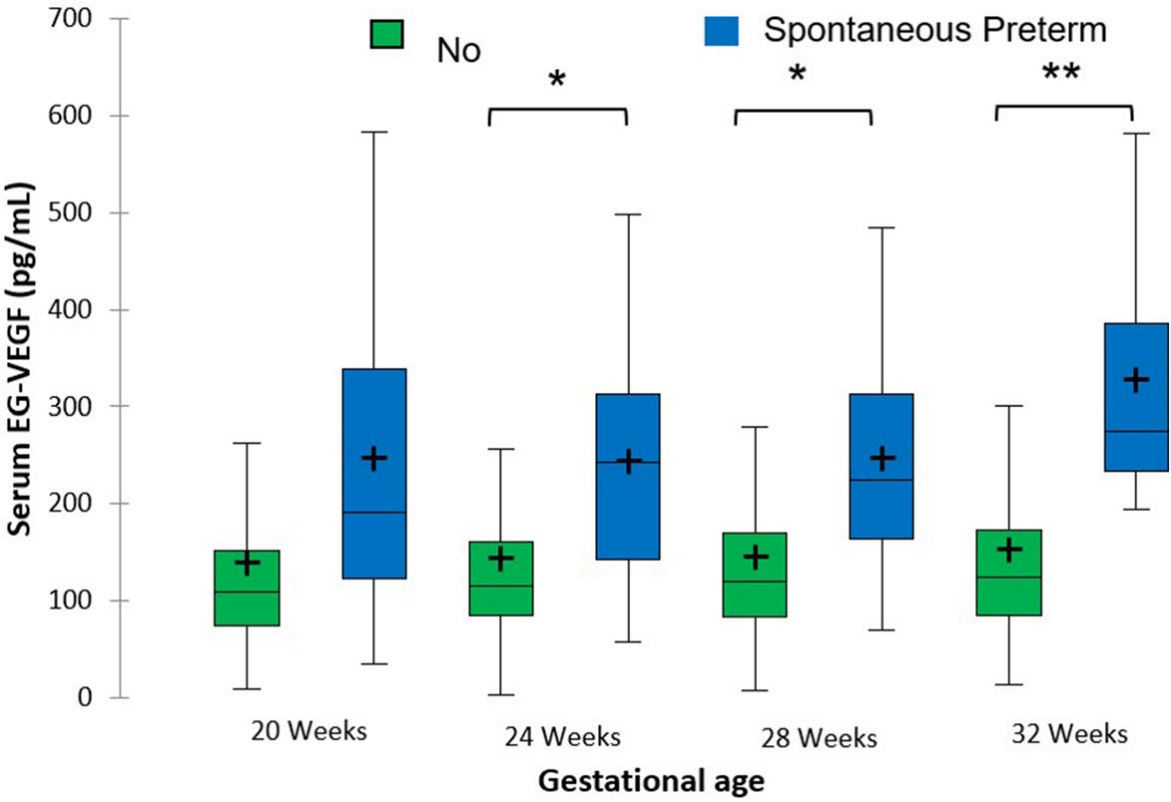
Circulating EG-VEGF concentrations at four gestational ages in uncomplicated and spontaneous preterm birth pregnant women. The central horizontal bars are the medians. The lower and upper limits of the boxes are the first and third quartiles. *p < 0.05 ** < 0.001.

Women with sPTB had higher concentrations of EG-VEGF than uncomplicated patients at 24 weeks (244.1 versus 144.3 pg/mL), 28 weeks (247.5 versus 146.2 pg/mL) and 32 weeks (328.5 versus 152.7 pg/mL) (p = 0.03, 0.02 and < 0.001). Results are summarized in Fig. 1.

**Figure 2:**
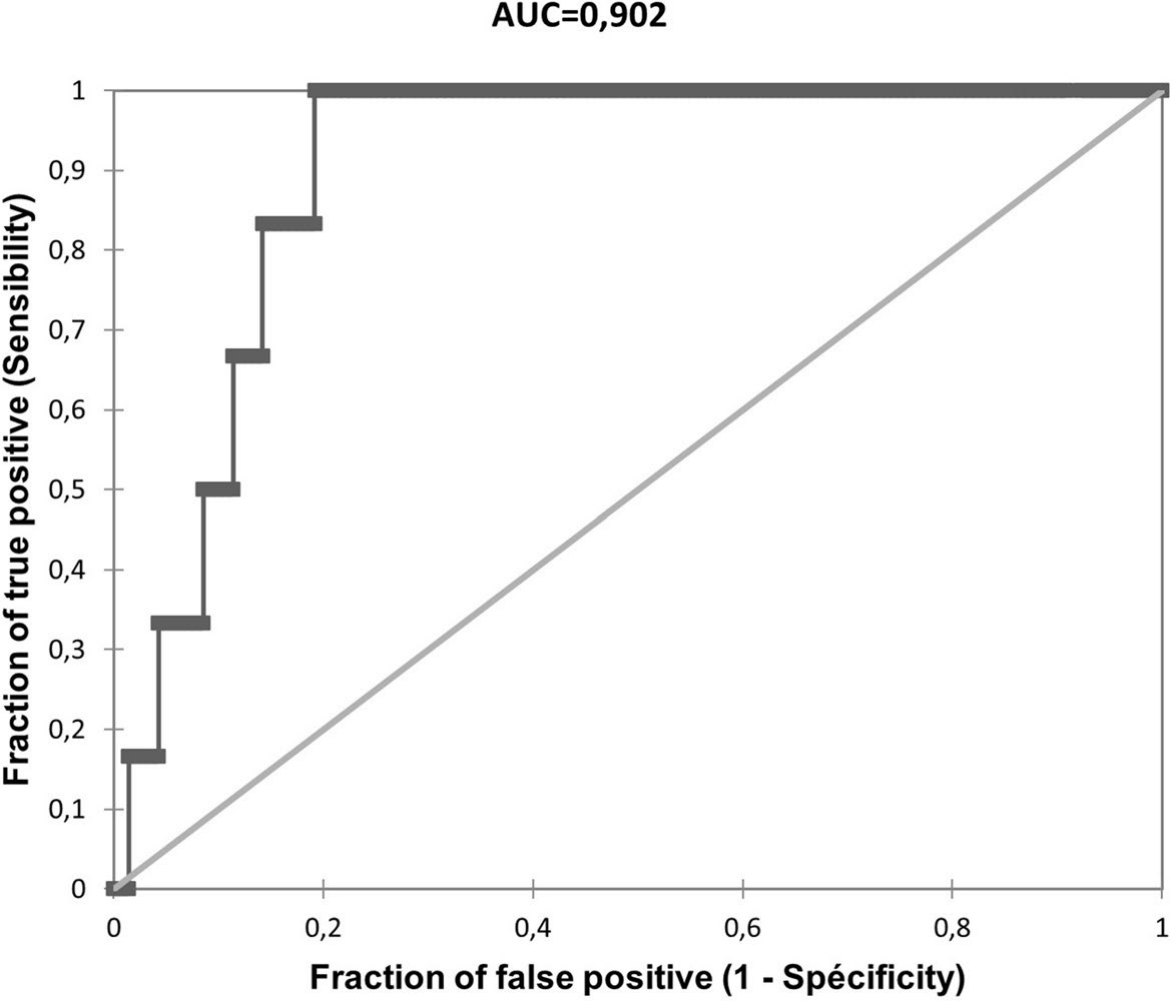
ROC curves analysis of circulating EG-VEGF concentrations for predicting spontaneous preterm birth at 32 weeks. *AUC* area under the curve.

ROC curves were used to calculate the threshold values of serum EG-VEGF levels to exhibit the best sensitivity and specificity for the prediction of sPTB (Table 2). The areas under the curve (AUC) reached 0.9 with 100% of sensitivity at 32 weeks for prediction of spontaneous preterm birth (Fig. 2).

**Table 2:** Performance of EG-VEGF for prediction of spontaneous preterm birth.

EG-VGEF cutoff (pg/mL)	Gestational age (weeks)	AUC	p value	Se	Sp	PPV	NPV
155.32	20	0.698	0.15	71.4	77.0	12.8	98.3
242.34	24	0.741	0.04	57.1	89.4	21.1	97.7
197.14	28	0.762	0.017	71.4	84.3	18.5	98.3
194.84	32	0.902	**< 0.001**	100	80.9	18.2	100

*AUC* area under curve, *Se* sensitivity, *Sp* specificity, *PPV* predictive positive value, *NPV* negative predictive value.

Significant values are in bold.

## Discussion

Serum EG-VEGF levels were predictive of spontaneous preterm birth as they were increased as early as 24 weeks of gestation. Circulating EG-VEGF exhibited higher levels in women at high risk for placenta-mediated complications with spontaneous preterm birth with a strong prediction capacity at 32 weeks.

Previous reports from our group demonstrated that in normal pregnancy, circulating EG-VEGF levels increased during the third trimester compared to the second trimester, but decreased during labor compared to patients with no labor^12^. EG-VEGF is also expressed in the mouse fetal membranes by the end of gestation, suggesting a local role for this protein in the mechanism of parturition^12^. One can speculate that EG-VEGF is a cytokine that may act locally to ensure fetal membranes protection in late pregnancy and that its decreased-expression may contribute to the initiation of the process of labor, as revealed by the abrupt decrease in its levels of expression as well those of its receptors.

So far, no study has assessed the prediction capacity of EG-VEGF level of spontaneous preterm birth. The increase in EG-VEGF levels in patient with sPTB as early as 24 WG substantiated our assumptions regarding the direct involvement of this factor in the etiology of pregnancy-associated pathologies^10,11,13–15,23^. A compensatory role of EG-VEGF in the etiology of sPTB is very likely and might involve reactivation of angiogenesis and control of labor-associated inflammation that are known to be controlled by this cytokine^12^.

Microbiological studies suggest that intrauterine infection may account for 25 to 40% of preterm births^24^. Importantly, Jabbour et al.^19^ tested the potential involvement of EG-VEGF in preterm induction upon its injection in animal models. EG-VEGF was compared to lipopolysaccharide (LPS), a component of the cell wall of the Gram-negative bacteria *Escherichia coli*, known to induce labor. LPS but not EG-VEGF injection induced preterm delivery within the following 20 h. EG-VEGF injection induced an increase in the mRNA expression of the pro-inflammatory mediators IL-6, IL-1, tumor necrosis factor (TNF), CXCL2, and CXCL5 within the cells of the fetal membranes^25^. EG-VEGF also increased the same inflammatory mediators in the human myometrium^17^. In addition, monocytes treated with EG-VEGF for 24 h released the chemokines CXCL1, CXCL8, and CCL4, and co-stimulation by LPS lead to a synergic effects on the production of CCL18 and CCL20^19^. While these findings strongly suggest that EG-VEGF is involved in the process that accompany preterm birth, to date we do not know whether its increased levels are cause or consequence of the preterm labor and whether EG-VEGF is a foe or friend of pregnancy pathologies. Further in vivo studies are ongoing to decipher the significance of EG-VEGF elevation 2–6 weeks before the occurrence of sPTB.

Altogether, our study demonstrates for the first time that EG-VEGF could be considered as a strong biomarker of the occurrence sPTB. This study completes the knowledge about biological markers which are associated with sPTB. Other biomarkers also seem interesting in recent literature like cellular inflammatory markers of activated macrophages^26–28^, the association of prenatal levels of proinflammatory C-reactive protein and interleukin-8^29^ and many others.

Our study have some limitations. It was not designed to explore spontaneous prematurity why we lack information on the precise context of prematurity apart from the term of birth and the reason for premature delivery. Another major limitation is the number of cases in the sPTB group. The strengths of this study are the examination of a population of patients at high risk of PMC who were recruited prospectively and followed from 20 weeks to delivery.

The association of EG-VEGF levels with spontaneous preterm birth will be investigated in larger cohorts to validate its informational value and propose it use in routine assessment of patient with high risk for placenta-mediated complications of this pathology.

## Data Availability

The datasets generated during and/or analysed during the current study are available from the corresponding author on reasonable request.
